# Microevolutionary traits and comparative population genomics of the emerging pathogenic fungus *Cryptococcus gattii*

**DOI:** 10.1098/rstb.2016.0021

**Published:** 2016-12-05

**Authors:** Rhys A. Farrer, Kerstin Voelz, Daniel A. Henk, Simon A. Johnston, Matthew C. Fisher, Robin C. May, Christina A. Cuomo

**Affiliations:** 1 Genome Sequencing and Analysis Program, The Broad Institute of MIT and Harvard, Cambridge, MA 02142, USA; 2 Department of Infectious Disease Epidemiology, School of Public Health, Imperial College London, London W2 1PG, UK; 3 Institute of Microbiology and Infection, School of Biosciences, University of Birmingham, Birmingham B15 2TT, UK; 4 NIHR Surgical Reconstruction and Microbiology Research Centre, University Hospitals Birmingham NHS Foundation Trust, Queen Elizabeth Hospital Birmingham, Birmingham B15 2TH, UK

**Keywords:** *Cryptococcus gattii*, microevolution, mitochondrial tubularization, intracellular proliferation

## Abstract

Emerging fungal pathogens cause an expanding burden of disease across the animal kingdom, including a rise in morbidity and mortality in humans. Yet, we currently have only a limited repertoire of available therapeutic interventions. A greater understanding of the mechanisms of fungal virulence and of the emergence of hypervirulence within species is therefore needed for new treatments and mitigation efforts. For example, over the past decade, an unusual lineage of *Cryptococcus gattii*, which was first detected on Vancouver Island, has spread to the Canadian mainland and the Pacific Northwest infecting otherwise healthy individuals. The molecular changes that led to the development of this hypervirulent cryptococcal lineage remain unclear. To explore this, we traced the history of similar microevolutionary events that can lead to changes in host range and pathogenicity. Here, we detail fine-resolution mapping of genetic differences between two highly related *Cryptococcus gattii* VGIIc isolates that differ in their virulence traits (phagocytosis, vomocytosis, macrophage death, mitochondrial tubularization and intracellular proliferation). We identified a small number of single site variants within coding regions that potentially contribute to variations in virulence. We then extended our methods across multiple lineages of *C. gattii* to study how selection is acting on key virulence genes within different lineages.

This article is part of the themed issue ‘Tackling emerging fungal threats to animal health, food security and ecosystem resilience’.

## 1. Introduction

Emerging fungal pathogens and fungal-like organisms are increasingly threatening natural populations of animals and plants [1]. For example, the recently discovered chytrid fungus *Batrachochytrium salamandrivorans* was implicated in the near extirpation of fire salamanders in 2013 in the Netherlands [2]. Race Ug99 of the basidiomycetous fungus *Puccinia graminis* f. sp*. tritici* first detected in 1998 is now recognized as a major threat to wheat production and food security worldwide [3], and the basidiomycetous fungus *Cryptococcus gattii* (*C. gattii*) has expanded its range into non-endemic environments with a consequential increase in fatal meningitis in humans [4,5]. The global threat of these and other related diseases is underpinned by fungi harbouring complex and dynamic genomes [6]. This leads to an ability to rapidly evolve in order to overcome host defences [7], presenting a pressing challenge to understand the mechanisms that drive the evolution of the phenotypic determinants that underlie pathogenicity.

*Cryptococcus gattii* causes pneumonia and meningoencephalitis in humans following an inhalation of infectious yeast or airborne hyphae [8]. While its sister species *Cryptococcus neoformans* is most prevalent in HIV-infected individuals and patients with other immunodeficiencies, *C. gattii* predominantly (although not exclusively; [9]) cause disease in healthy people [10]. *C. gattii* accounts for less than 1% of all cryptococcosis cases and until the late 1990s was found mostly in tropical and subtropical parts of the world. However, in 1999, an outbreak of *C. gattii* was reported on Vancouver Island in domestic pets, wild animals and people [4,11]. This outbreak spread to mainland Canada and then into the Northwestern states of the USA and remains a major public health concern.

*Cryptococcus gattii* is divided into four distinct lineages (VGI, VGII, VGIII and VGIV), with such considerable genetic variation that they were recently described as separate species (*C. gattii*, *C. deuterogattii*, *C. bacillisporus* and *C. tetragattii,* respectively [12]). VGI and VGII isolates are responsible for the majority of infections in immunocompetent individuals in the Pacific Northwest, the North of Australia and in Central Papua New Guinea [13]. Although the original outbreak on Vancouver Island was caused by at least two clonal subgroups of VGII named VGIIa (the major genotype) and VGIIb (the minor genotype) [11], several associated outbreaks have subsequently been reported, e.g. VGIIc in Oregon, USA [14]. Recent studies investigating the genetic diversity of outbreak isolates by whole genome sequence typing have identified an abundance of genetic diversity within the VGII molecular type and evidence for both sexual recombination and clonal expansions [15–17].

The ability of cryptococcal cells to parasitize phagocytes, in particular macrophages, is a major pathogenesis mechanism of cryptococcosis [18,19]. *C. gattii* is able to protect itself from host-induced oxidative stresses, such as reactive oxygen species (ROS), via an enlarged polysaccharide capsule, which provides a physical barrier that interferes with normal macrophage phagocytosis and clearance by the immune system [20]. Although all four lineages are capable of causing disease, a number of differences have been identified between sublineages, such as increased intracellular proliferation rates (IPRs) in VGIIc isolates [5], or an enhanced ability to parasitize host phagocytic cells by VGIIa outbreak isolates. These processes are initiated upon engulfment by macrophages, followed by a stress response that triggers cryptococcal mitochondrial tubularization and rapid proliferation of the outbreak strains [19]. Another study identified increased expression levels for laccase in the VGIIa isolate R265 compared with non-outbreak strains; laccase controls melanin production and provides protection from oxidative damage imposed by the host immune response. In addition, cryptococcal strains are able to escape phagocytes by a non-lytic mechanism (expulsion or ‘vomocytosis’ [21,22]) or to undergo ‘lateral transfer’ between phagocytes. These processes may provide greater resistance to stresses in the phagosome and may also have a role in the dissemination of the pathogen from the lungs to the central nervous system.

Genomic comparisons between lineages have identified a range of genetic differences that may contribute to differences in fitness, ranging from chromosome copy number variation to genomic rearrangements [23,24]. Furthermore, as many as 700 genes are unique to one or more of the four lineages, including heat-shock proteins and iron transporters, which could contribute to differences in disease progression and outcome [24]. Positive selection has also been identified among orthologous multi-drug transporters in different lineages and clonal groups [24]. However, new emerging or hypervirulent genotypes arise at the population level, and may not be detected from comparisons between more anciently diverged isolates. Here, we combine phenotypic typing from 20 *C. gattii* strains from each of the four lineages, with a case-study genomic comparison for two highly genetically similar isolates belonging to VGIIc (EJB18 and EJB52) that have marked differences in intracellular proliferative rates (IPRs) and mitochondrial tubularization rates. Our approach identifies 33 candidate nuclear genes that may contribute to these hypervirulent traits. Finally, we extend our approach to study the wider population structure and variation among a panel of 64 *C. gattii* isolates and demonstrate how the methods we detail here are applicable to investigating the genetic determinants that underpin virulence across a wide range of emerging fungal pathogens.

## 2. Results

### (a) Whole genome sequencing and phenotypic typing suggest a loss/gain pattern of hypervirulence traits among VGIIc

**Figure 1.**
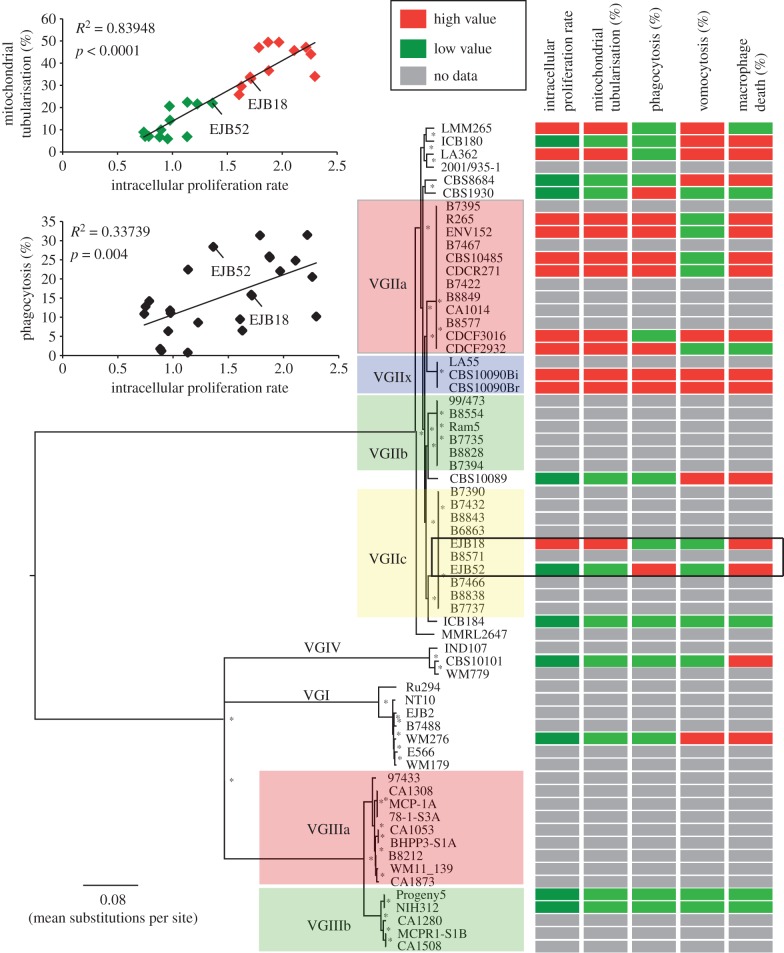
Correlation of phylogenetic and phenotypic data. Phenotypic data were superimposed onto the phylogenetic reconstruction of the nuclear genome and the data clustered into high- and low-value groups using a *k*-means clustering approach. The abilities to proliferate within macrophages and to form tubular mitochondria upon engulfment are strong virulence markers. (Top left) Mitochondrial tubularization and yeast uptake by macrophages were correlated (*p* < 0.0001 and *p* = 0.004, respectively) with their intracellular proliferative rate (IPR). Asterisks indicate 100% bootstrap support from 1000 replicates, and a box is used to highlight the VGIIc isolates that have shifts in phenotype.

Detecting microevolutionary changes requires precision variant-calling to distinguish subtle differences with sequencing or alignment error. Using a highly stringent SNP-calling protocol (see §4b), we were able to reduce false-positive SNPs to 4.5 per one million bases (*n* = 77; 0.13% of all SNPs called), while maintaining 99.3% true-positive SNPs (the remaining 0.7% were false-negatives). Applying these parameters to 64 isolates of *C. gattii* (table 1 and electronic supplementary material, table S1), we identified SNPs in all isolates and used these to construct a phylogenetic tree, illustrating the sublineages within each of the four major lineages of *C. gattii* (figure 1).

**Table 1.** *Cryptococcus gattii* strains included in this study.

strain type	strain	origin	est. lat.	est. long.	source	mating type
VGI	B7488	USA, Oregon	43.8	−120.5	clinical	alpha
VGI	E566	South Australia	−25.5	134.0	eucalyptus tree	a
VGI	EJB2	USA North Carolina, with a history throughout the San Francisco, CA	46.0	−121.0	clinical	alpha
VGI	NT-10	Australia	−25.5	134.0	clinical	alpha
VGI	Ru294	South Africa	−31.0	23.7	unknown tree	alpha
VGI	WM179	Sydney, Australia, 1993	−33.9	151.1	clinical	alpha
VGI	WM276	Australia	−25.5	134.0	environmental	alpha
VGII	2001/935	Senegal	14.2	−14.4	clinical	alpha
VGII	99/473	Caribbean Islands	20.8	−77.6	clinical	alpha
VGII	CBS10089	Greece	39.5	21.8	clinical	alpha
VGII	CBS1930	Aruba, Caribbean Sea	12.5	−70.0	goat	a
VGII	CBS8684	Uruguay	−32.9	−56.0	environmental (wasp nest)	alpha
VGII	ICB180	Sao Paulo, Brazil	−9.5	−55.8	environmental (eucalyptus tree)	alpha
VGII	ICB184	Piaui, Brazil	−9.5	−55.8	environmental (tree)	alpha
VGII	LA362	Brazil, Jaboticabal	−21.3	−48.3	animal (Parrot lier?)	alpha
VGII	LMM265	Brazil	−9.5	−55.8	clinical	alpha
VGII	MMRL2647	Caribbean Islands	−25.5	134.0	clinical	alpha
VGIIa	B7395	USA, Washington	38.9	−77.0	dog	alpha
VGIIa	B7422	USA, Oregon	43.8	−120.5	cat	alpha
VGIIa	B7467	USA, Washington	38.9	−77.0	porpoise	alpha
VGIIa	B8577	Canada, British Columbia	53.9	−127.6	environmental	alpha
VGIIa	B8849	USA, Oregon	43.8	−120.5	environmental	alpha
VGIIa	CA1014	USA	46.0	−121.0	clinical	alpha
VGIIa	CBS10485	Canada, Vancouver Island	49.7	−125.2	clinical (Danish tourist)	alpha
VGIIa	CDCF2932	Canada, British Columbia, Kelowna	49.9	−119.5	clinical (immunocompetent patient)	alpha
VGIIa	CDCF3016	Canada, shores island close to Vancouver Island	49.7	−125.2	animal (dead wild dall's porpoise)	alpha
VGIIa	CDCR271	Canada, British Columbia, Nanoose Bay	49.3	−124.2	clinical (immunocompetent male)	alpha
VGIIa	ENV152	Canada, Vancouver Island, Provincial Park, Rathrevor Beach	49.3	−124.3	environmental (alder tree)	alpha
VGIIa	R265	Canada, British Columbia, Duncan	48.8	−123.7	clinical	alpha
VGIIb	B7394	USA, Washington	38.9	−77.0	cat	alpha
VGIIb	B7735	USA, Oregon	43.8	−120.5	clinical	alpha
VGIIb	B8554	USA, Oregon	43.8	−120.5	dog	alpha
VGIIb	B8828	USA, Washington	38.9	−77.0	porpoise	alpha
VGIIb	Ram5	Australia	−25.5	134.0	clinical	alpha
VGIIc	B6863	USA, Oregon	43.8	−120.5	clinical	alpha
VGIIc	B7390	USA, Idaho	44.2	−114.8	clinical	alpha
VGIIc	B7432	USA, Oregon	43.8	−120.5	clinical	alpha
VGIIc	B7466	USA, Oregon	43.8	−120.5	cat	alpha
VGIIc	B7737	USA, Oregon	43.8	−120.5	clinical	alpha
VGIIc	B8571	USA, Washington	38.9	−77.0	clinical	alpha
VGIIc	B8838	USA, Washington	38.9	−77.0	clinical	alpha
VGIIc	B8843	USA, Oregon	43.8	−120.5	clinical	alpha
VGIIc	EJB18	USA, Oregon	43.8	−120.5	clinical	alpha
VGIIc	EJB52	USA, Oregon	43.8	−120.5	clinical	alpha
VGIIIa	78-1-S3A	Los Angeles, California, USA, 2011	34.0	−118.3	environmental	alpha
VGIIIa	97/433	Mexico	23.4	−101.7	clinical	alpha
VGIIIa	B8212	USA, Oregon	43.8	−120.5	unknown	alpha
VGIIIa	BHPP3-S1A	Los Angeles, California, USA, 2012	34.0	−118.3	environmental, soil	alpha
VGIIIa	CA1053	California, USA	36.5	−119.7	clinical	alpha
VGIIIb	CA1280	USA	46.0	−121.0	clinical	alpha
VGIIIa	CA1308	California, USA	36.5	−119.7	clinical	alpha
VGIIIb	CA1508	California, USA	36.5	−119.7	clinical	a
VGIIIa	CA1873	USA	46.0	−121.0	clinical	a
VGIIIa	MCP-1A	Los Angeles, California, USA, 2011	34.0	−118.3	environmental	alpha
VGIIIb	MCPR1-S1B	Los Angeles, California, USA, 2012	34.0	−118.3	environmental, soil	a
VGIIIb	NIH312	California, USA	36.5	−119.7	clinical	alpha
VGIIIb × VGIIx	Progeny5	n.a.	n.a.	n.a.	n.a.	alpha
VGIIIa	WM11_139	USA, 2011	43.8	−120.5	veterinary	a
VGIIx	CBS10090_Bir	Greece	39.5	21.8	clinical	a
VGIIx	CBS10090_Bro	Greece	39.5	21.8	clinical	a
VGIIx	LA55	NE region of Piaui, Brazil	−9.5	−55.8	clinical	a
VGIV	CBS10101	South Africa	−31.0	23.7	king cheetah	alpha
VGIV	IND107	India	22.4	78.9	clinical	alpha
VGIV	WM779	South Africa, 1994	−31.0	23.7	veterinary	alpha

Macrophage parasitism and the ability to proliferate within these phagocytic effector cells are well-established virulence traits of cryptococcal infections. To correlate genetic and phenotypic distance, we measured a range of macrophage interaction parameters (i.e. phagocytosis, IPR, vomocytosis, cryptococcal mitochondrial tubularization and macrophage death) in replicate (3X–8X) over a timecourse of macrophage interaction (0, 18, 24 and 48 h). First, we measured the maximal intracellular proliferate potential (*T*_max_; commonly referred to as IPR) for 20 isolates of *C. gattii* including four of the six named subclades (the outbreak clades VGIIa and VGIIc, the recently described VGIIx [24], and VGIIb; figure 1 and electronic supplementary material, table S2). These strains were selected, according to previous literature and strain detail knowledge, to represent a balanced collection of strains (i) from the North Pacific *C. gattii* outbreak, (ii) from environmental origin, and (iii) representing the different molecular groups. IPR values ranged from 0.74 to 2.30, and *K*-means clustering revealed two groups. One group contained isolates with lower values found across all of the lineages and therefore not correlated with phylogeny (all less than 1.5 IPR). These included one VGI isolate, five of the seven non-clonal subclades of VGII, both VGIIIb isolates and one VGIV isolate. In contrast, all six of the VGIIa isolates and the VGIIx isolate were in the cluster with higher values (greater than 1.7 IPR).

In addition to IPR, we measured host–pathogen interactions via the induction of mitochondrial tubularization (specifically, an average per cent of yeast with tubular mitochondria), which is associated with response to, and protection from, ROS upon engulfment [25]. Again, values were highly variable among the four lineages (electronic supplementary material, table S2), with the highest proportion of tubularizing mitochondria among the subclades of VGII. Intracellular proliferative capacity correlated significantly with mitochondrial tubularization and yeast uptake by macrophages (*p* < 0.0001 and *p* = 0.004, respectively, figure 1) [25]. Of note, two VGIIc isolates show large differences in mitochondrial tubularization (EJB52 = 18% and EJB18 = 44%) and IPR (EJB52 = 1.36 ± 0.33 IPR and EJB18 = 1.71 ± 0.35 IPR), suggesting that these isolates are suitable for further investigation. Substantial variation was also observed in the fraction of yeast phagocytized, with uptake per cent varying from 0.5% to 31.5% (figure 1 and electronic supplementary material, table S2). The greatest per cent uptakes were from most of VGIIa, VGIIx and importantly VGIIc EJB52 (28.4%), whereas VGIIc EJB18 was phagocytized less (15.7%).

We also assayed each isolate for the rate of expulsion after phagocytosis by macrophages (‘vomocytosis’) and macrophage cell death. In contrast to IPR, mitochondrial tubularization and percentage phagocytosis, there was far lower intralineage variation in non-lytic escape (‘vomocytosis’) and in host cell death (figure 1 and electronic supplementary material, table S2). Furthermore, neither of these two phenotypic markers significantly correlated (*p* = 0.837 and *p* = 0.235, respectively) with IPR rate. This may be an indication that *C. gattii* hypervirulence is driven by the correlated phenotypes and not by ‘vomocytosis’ or macrophage death.

Comparing closely related isolates for shifts in phenotypes (the pattern of IPR, mitochondrial tubularization and phagocytosis) shows the greatest discrepancy between the two VGIIc isolates EJB52 and EJB18. A two-tailed *t*-test of the IPR replicates also suggested a difference in values (*p*-value = 0.0458). The limited genetic variation and large phenotypic difference between these two isolates suggested they were good candidates to identify the genetic differences that may be responsible.

To determine if phenotypic variation in VGIIc stemmed from gene loss, we measured read coverage and depth across each gene. Read depth revealed a total of 686 presence/absence (P/A) polymorphisms in at least one isolate, of which 16 were absent in both VGIIc isolates (electronic supplementary material, table S3). No P/A polymorphisms were found in VGIIa isolates to which the reference R265 belongs. Although the 16 P/A polymorphisms in VGIIc could not explain the phenotypic differences between the two VGIIc isolates, they may be relevant to phenotypic differences between subclades (such as between VGIIa and VGIIc). Three of the VGIIa genes absent in VGIIc have both zinc-binding dehydrogenase and alcohol dehydrogenase GroES-like PFAM domains. These are likely the most abundant zinc-binding proteins in the cell, and may play a role during zinc-deprivation conditions such as within a phagosome [26]. Two additional proteins had major facilitator superfamily and sugar transport PFAM domains, which may play out as differences in the ability to transport toxins or xenobiotics out of the cell, or transport sugars into the cell, respectively.

**Figure 2.**
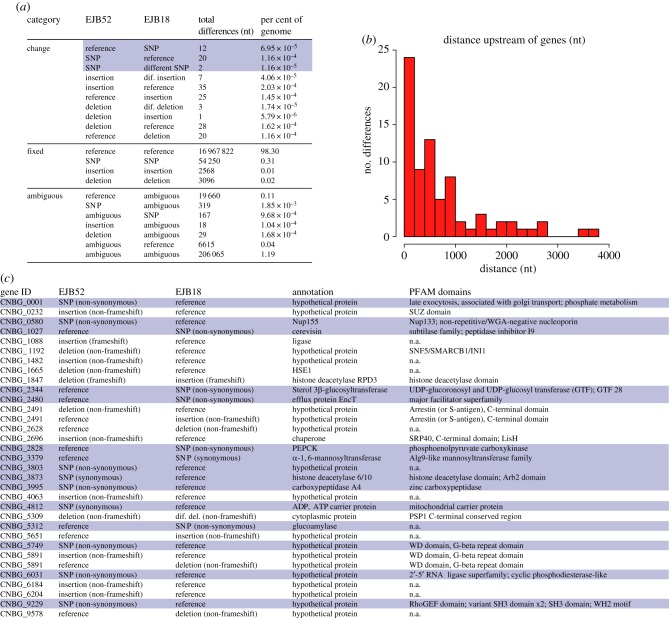
Genetic changes that underlie the increased hypervirulence of *C. gattii* outbreak strains were identified by comparing EJB52 (low per cent mitochondrial tubularization and IPR) and EJB18 (high tubularization and IPR). (*a*) Summary of all genetic differences between EJB52 and EJB18. Single base changes are shown in blue (*b*) Distance of intergenic variants between EJB52 and EJB18 to any upstream genes. (*c*) Thirty-one genes with genetic differences were uniquely identified between EJB52 and EJB18. Genes are numerically ordered by their locus ID, and single base changes are highlighted. (Online version in colour.)

We classified every base of the 17.3 Mb genomes of EJB18 and EJB52, placing 98.65% into a non-ambiguous subcategory (the remaining 1.35% of the genomes were ambiguous, i.e. poorly supported base call in one or both of the two isolates). Nucleotide subcategories were annotated in a codon-by-codon manner, as either fixed or transitional between the two isolates. Only 153 differences (8.9 per Mb; figure 2*a* and electronic supplementary material, table S4) were identified between these isolates (compared with 60 thousand; 3.5 per kb from their initial alignments to the VGIIa R265 reference genome; electronic supplementary material, table S1). Importantly, no differences were detected within the mitochondrial genome, suggesting that the phenotypic differences in the regulation of mitochondrial tubularization are encoded in the nuclear genome. Furthermore, no aneuploidy was detected based on depth of coverage plots.

Of the 153 sites differing between EJB18 and EJB52, a subset of 33 variants overlapped sites among the 6456 predicted genes of R265, including 15 SNPs and 18 indels (figure 2*c*). The remaining differences fell within introns (*n* = 32) and intergenic (*n* = 88) regions, differences that could also have an impact if they, for example, fell within a promoter or repressor region. To examine this, we identified all intergenic differences that were upstream of a gene (figure 2*b*), 13 of which were within 100 nt. The closest upstream intergenic difference was an insertion unique to EJB52, 12 bases upstream of the STE/STE11/SSK protein kinase (CNBG_4621), involved in *Cryptococcus* mating and virulence [27]. Separately, a solute carrier family 25 (mitochondrial citrate transporter) gene had two upstream intergenic differences: an insertion 46 nt upstream unique to EJB52 and a deletion 44 nt upstream unique to EJB18. Improper uptake or conversion of citrate is known to attenuate a range of pathogens, including *Cryptococcus* [28–30].

Of the 31 genes that had differences between EJB52 and EJB18 (figure 2*c*; two genes had two differences), two were synonymous changes, and eight were hypothetical proteins without any assigned functional information (GO-terms, PFAM, TIGRFAM). The remaining 23 genes had non-synonymous differences and functional annotation. As mitochondrial tubularization is one of the main phenotypic differences between the two VGIIc isolates, we predicted proteins that are localized to the mitochondria. Of the 6456 proteins in *C. gattii* VGII, only 548 were predicted to localize to the mitochondria; two of these genes had genetic differences between the two VGIIc isolates. The first gene (CNBG_5651) has an insertion (non-frameshift/modulo 3) in EJB18 (high value tubularization), and its specific function is unclear. Furthermore, this allele is not correlated with the mitochondrial tubularization phenotype across the isolates as the insertion was found in 40 of 66 isolates including both high and low mitochondrial tubularization percentages. The second gene (CNBG_5312) has a non-synonymous change (T → C, I → V) in glucoamylase in EJB18; this change is unique to EJB18. Glucoamylase expression is responsible for carbohydrate metabolism in other intracellular pathogens, such as *Listeria pneumophila* within amoebae [31,32], and *C. gattii* is also likely evolving for protection against this [33].

Although not predicted to localize to the mitochondria, a gene with the mitochondrial carrier protein domain (CNBG_4812) has a unique synonymous SNP in EJB52 (low tubularization) compared with EJB18; this change also does not fall within a splice donor/acceptor site. Curiously, this SNP was also present in six of the seven VGI isolates sequenced, including WM276 that also shows low tubularization. The other strains with this change were not measured for this phenotype, including the six other VGI isolates and one other VGIIc isolate (B7466). Finally, it is noteworthy that two separate histone deacetylases vary between the isolates, including a synonymous SNP unique to EJB52 in CNBG_3873 annotated as ‘6/10/Arb2’ and a deletion in EJB52→insertion in EJB18 for CNBG_1847 annotated as ‘RPD3’. The deletion was found in only one other isolate: the closely related VGIIc B7466, for which we have no phenotypic data. However, it is possible that unique variants give rise to shared phenotypes by disrupting the same gene or gene network. For example, histone deacetylases are involved in the morphology and virulence of *Candida albicans* [34].

### (b) Selection in the clonal groups of *Cryptococcus gattii* acts upon capsule genes, heat-shock proteins and the STE/STE11/SSK protein kinase

**Figure 3.**
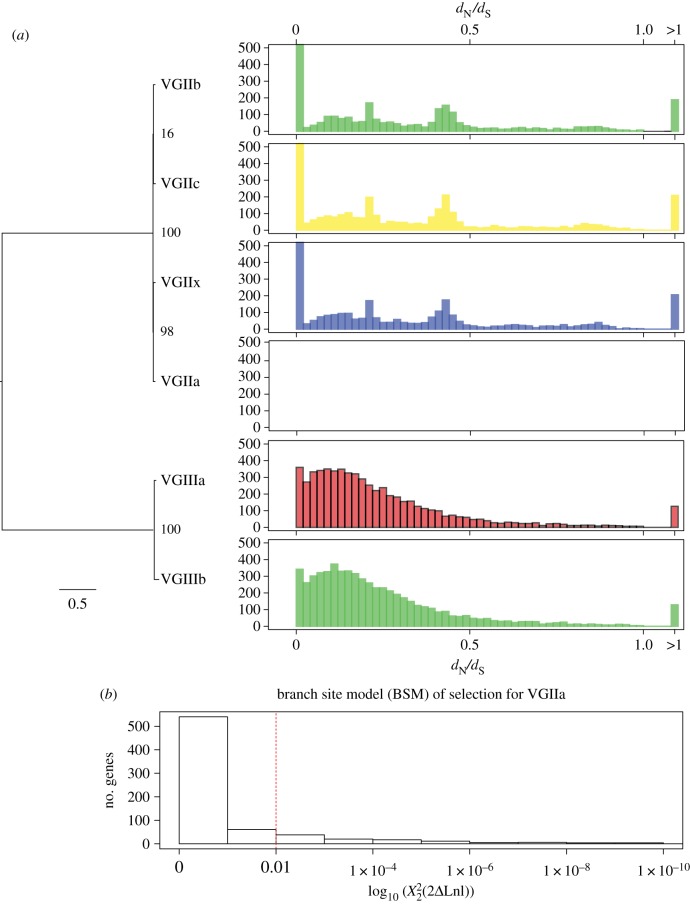
Phylogenetic relationships and selection of the *C. gattii* clonal subclades. (*a*) A RAxML tree with the GTRCAT model and 1000 bootstrap replicates next to a histogram showing the number of genes with binned *d*_N_/*d*_S_ (*ω*) values. (*b*) Histogram of 714 genes with 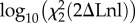 values from the branch site model (BSM) of selection in PAML. The remaining genes did not have values (e.g. owing to insufficient genetic distance). The dashed red line is at *q* = 0.01, which we have used as a cut-off for significance. (Online version in colour.)

To examine the impact of selection on the different *C. gattii* clades, we measured *d*_N_/*d*_S_ (*ω*) values for fixed differences across six subclades of *C. gattii* (2a, 2b, 2c, 2x, 3a, 3b). A total of 859 genes have *d*_N_/*d*_S_ with values greater than 1, which can be indicative of relaxed or positive selection (figure 3). No PFAM or GO-term from these genes was enriched (according to two-tailed Fisher's exact test with *q*-value false-discovery rate (FDR) against the remaining gene-sets). However, two genes of known interest were identified. The first gene (CNBG_1370) is a 1 : 1 orthologue of *C. neoformans* H99 Utr2 gene (with homology to chitin transglycolase), which is potentially involved in capsule biosynthesis [35]. In *C. gattii,* this gene is under selection in the recently named VGIIx subclade [24] with *d*_N_ = 0.0063, *d*_S_ = 0.0059, *ω* = 1.0792. The second gene (CNBG_0047) is in an orthogroup with *C. neoformans* H99 Cap64-like proteins Cas31 and Cas3, and under selection in VGIIb (*d*_N_ = 0.0028, *d*_S_ = 0.0023, *ω* = 1.2242). Both Cas31 and Cas3 mutants have decreased capsule sizes in *C. neoformans*, as they are involved in determining the position and the linkage of the xylose and/or *O*-acetyl residues on the mannose backbone of the capsule [35,36].

The reference isolate VGIIa R265 is only separated from other VGIIa isolates by between 39 and 184 SNPs (table S1), of which only 12 were fixed (i.e. likely errors in the R265 assembly itself). To measure selection in VGIIa, we therefore used the branch site model (BSM) of selection implemented by PAML [37]. We found 113 genes with significant differences (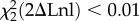) after Benjamini–Hochberg (BH) multiple correction. Previously when we calculated these values for VGIIa (subset 5 in [24]) for non-fixed differences across multiple isolates with BH correction and *q*-value < 0.01, without adjusting the NS sites parameter, we identified an almost entirely distinct list of 87 genes, apart from two genes that were identified in both (CNBG_5460 (Fungal Zn(2)-Cys(6) binuclear cluster domain) and CNBG_4219 (domain of unknown function 1708)). Again, no PFAM or GO-term from these genes was enriched (according to two-tailed Fisher's exact test with *q*-value FDR). However, we did identify a transcription factor from the Zn(2)-Cys(6) family named CTA4 that is a nitric oxide-responsive element, and required for the nitrosative stress response in *C. albicans* [38].

Other important genes found to be under selection in the VGIIa branch using the BSM included a heat-shock protein 71 (CNBG_5963) and an ABC transporter (MDR/TAP) member 1 (CNBG_9005), both of which are known to be involved in virulence by a range of pathogens [39–42]. Selection was found in the VGIIa branch for the STE/STE11/SSK protein kinase (CNBG_4621), which was also identified and discussed earlier for having the closest (12 nt) upstream intergenic difference between VGIIc isolates EJB52 and EJB18. Finally, an orthologue to the *C. neoformans* H99 capsule gene Pmt4 (*C. gattii* gene CNBG_0576) was found to be under selection (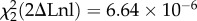)—specifically on a histidine at position 264. Furthermore, *C. neoformans* Pmt4 mutants have decreased capsule sizes [35]. Therefore, at least three of the four clonal subgroups of *C. gattii* VGII (a, b and x) have evidence for selection within microevolutionary timescales in one of their capsule biosynthesis genes.

## 3. Discussion

The aetiological agents of infectious disease impose a huge burden on human society. By understanding their biology, reproduction and mechanisms of infection, we are able to assess and discover new strategies for mitigating their impact. In recent years, fungi have gained widespread attention for their ability to rapidly emerge and threaten both animal and plant species across a global scale [1]. However, many features of their genomes that enable them to successfully adapt to infect diverse hosts and inhabit a wide range of ecological niches remain cryptic [6], especially for newly evolved emerging lineages. The underlying mechanism driving outbreaks caused by *C. gattii* has been puzzling researchers for over a decade. Compounding this is the fact that its virulence is likely a consequence of adaptations that have evolved for protection against environmental predators such as amoebae [33] (unless it is surviving and escaping out of dead animals).

Here, we have combined whole genome sequencing with phenotypic analysis to identify recent genetic changes that might underpin cryptococcal hypervirulence. Our phenotypic analysis demonstrated that IPR and mitochondrial tubularization, but not phagocytosis or expulsion by the host, correlate strongly with hypervirulence. This finding was unexpected, and may be informative for their predictive potential in future studies working with these phenotypes. One possible explanation is that there is a set of general virulence factors that allow both *C. neoformans* and *C. gattii* to establish within the human host. However, in the *C. gattii* hypervirulent strains, only the subset responsible for IPR and mitochondrial tubularization provide enhanced parasitism of innate immune effectors. Thus, our study supports previous findings that indicate the importance of IPR in cryptococcal virulence and suggests that IPR and mitochondrial tubularization may be useful as ‘proxy’ markers of virulence. We also found that these features can be surprisingly variable, even within very closely related isolates of a given subclade, such as those between EJB52 (low tubularization and IPR) and EJB18 (high tubularization and IPR) of VGIIc.

No differences were found between the mitochondrial genomes of the VGIIc isolates EJB52 and EJB18, suggesting that differences in their mitochondrial tubularization stems from differences in their nuclear genome—which already is thought to control mitochondrial function, fusion and fission [43]. In contrast, comparing the nuclear genomes of the VGIIc isolates EJB52 and EJB18 revealed a set of 31 genes that might be involved in the differences we found in cryptococcal hypervirulence. It is unlikely that all of these loci will be directly involved in virulence regulation. However, one or more of these genes are clear candidates, such as the two genes that localize to the mitochondria, including the glucoamylase or the two histone deacetylases. Although the variants were not correlative across the species at large, there may be numerous unique genetic routes to similar phenotypes. Some of the phenotypic differences may also be explained by non-direct, and/or epistatic, pleiotropic or epigenetic means. Potentially non-direct acting variants were identified within the intergenic regions, sometimes falling close upstream of transcription start sites, such as the insertion unique to EJB52 immediately upstream of the STE11/SSK protein kinase. That this gene appears to be under selection in the sister subclade VGIIa suggests that this is at least a region of dynamic variation, if not potentially involved in some phenotypic differences between these isolates.

The approach we have taken in this study is to combine phenotypic screening with whole genome comparisons in an attempt to identify the genomic determinants underpinning fungal virulence. To reduce the impact of sequencing errors, assembly errors in the reference sequence, alignments errors and variant call errors, it is essential to assess false-discovery rates and respond to those sources of error in an iterative approach [44]. In this study, we have identified 153 high confidence genetic differences that could explain differences in virulence traits between isolates. Detection of microevolutionary events can ascribe new mechanisms behind increased virulence, such as the increased expression of FRE3-encoded iron reductase in *C. neoformans* passaged in mice [45], or a single de novo heterozygous position within a gene called SSN3 capable of restoring filamentation in a non-filamentous *C. albicans* mutant [46].

The mechanisms behind changes in mitochondrial tubularization and IPR, which appear to be linked, may be the result of one or more genetic differences between high- and low-value isolates (such as those identified here). The number of genetic differences is sufficiently small that hypotheses can be generated and experimentally validated via gene disruptions or allele-swaps [47]. Alternatively, large panels of isolates can be screened via a genome-wide association study approach [48]. Where protein structures have been resolved, and are available, sites of positive selection are often in regions at the host–pathogen interface [49], further guiding a functional prediction. Finally, upstream variants that impact expression levels could be characterized using RNA-Seq. Ultimately, progress in pathogenomics heavily relies on open access and usability of well-maintained databases of sequence data, functional information and annotation, and pathogen-specific online resources for community-driven efforts.

We complimented the comparison of two closely related isolates with selection analysis across fixed variants, for which *d*_N_/*d*_S_ ratios were originally developed [50]. By focusing only on fixed differences, we found evidence for selection in capsule biosynthesis genes in each of the other three subclades of VGII, each of which had been phenotyped via mutants as having an effect on capsule size or likely to be involved in its assembly. Selection across these genes suggests that each subclade of VGII is generating new alleles and variation within the capsule genes, some of which may result in new peaks in an adaptive landscape, and become a distinguishing genetic feature of their clonal expansion.

## 4. Material and methods

### (a) Yeast and mammalian cells, growth conditions and phenotypic analysis

Twenty of the 64 *C. gattii* strains (table 1; SRP017762) typed in this study were cultured in liquid or agar YPD media (1% peptone, 1% yeast extract, 2% D-(+)-glucose) for 24 h at 25°C rotating at 20 rpm [13,14,19,25,51] prior to experiments. Mammalian J774 macrophage-like cells were grown as described previously [13,14,19,25,51].

Macrophages were infected with yeast cells and IPR monitored as previously described [13,14,19,25,51]. Cryptococcal mitochondrial morphology was determined as described previously [5]. We would like to note that some of the IPR and mitochondrial tubularization data were previously presented (see [25]), although this paper included additional biological repeats. For analysis of vomocytosis and macrophage cell death of sequenced strains, time-lapse images were captured on a TE2000 (Nikon) enclosed in a temperature-controlled and humidified environmental chamber (Okolabs) with 5% CO_2_ at 37°C with Digital Sight DS-Qi1MC camera (Nikon), 20× objective (Ph1 PLAN APO), using NIS elements AR software (Nikon). Images were captured every 2 min for 24 h. Vomocytosis (non-lytic expulsion of intracellular cryptococci) and infected macrophage cell death (disintegration of macrophage containing one or more cryptococci) were scored blind from four separate experiments for each of the 20 strains. Clusters of phenotypes were inferred via k-means clustering in R (kmeans) with 1000 iterations.

### (b) Variant-calling and sequence analysis

Alignment and SNP-calling parameters were initially optimized. The recently updated [24] nuclear and mitochondrial genome sequences and feature files for *C. gattii* isolate VGIIa R265α were used (GenBank project accession number AAFP01000000). Additional isolates sequenced and described in previous studies [16,24,52] were obtained from the short read archive (SRA) and converted from SRA format to FASTQ using the SRAtoolkit v. 2.3.3–4. Illumina reads were aligned to the genome sequence using Burrows–Wheeler aligner v. 0.7.4 mem [53] with default parameters to obtain high depth alignments (average 116X), and converted to pileup format using Samtools v. 0.1.18 [54]. To act as a control for sequencing, alignment and SNP-calling, we included the reference strain R265 in our panel of isolates.

The genome analysis toolkit (GATK) [55] v. 2.7-4-g6f46d11 was used to call both variant and reference bases from the alignments. First, the Picard tools AddOrReplaceReadGroups, MarkDuplicates, CreateSequenceDictionary and ReorderSam (see http://broadinstitute.github.io/picard) were used to preprocess the alignments. We used GATK RealignerTargetCreator and IndelRealigner for resolving misaligned reads close to indels on parental–progeny pairs of isolates to avoid discrepancies between isolates. Next, GATK UnifiedGenotyper (with haploid genotyper ploidy setting) was run with both SNP and INDEL genotype likelihood models (GLMs). We additionally ran BaseRecalibrator and PrintReads for base quality score recalibration on those initial sites for GLM SNP and then re-called variants with UnifiedGenotyper (emitting all sites). We next merged and sorted all of the calls, and ran VariantFiltration with the parameters ‘QD < 2.0, FS > 60.0, MQ < 40.0’. Next, we removed any base that had less than a minimum genotype quality of 50, or a minimum depth of 10. Finally, we removed any positions that were called by both GLMs (i.e. incompatible indels and SNPs), any marked as ‘LowQual’ by GATK, nested indels, or sites that did not include a PASS flag.

To assess the ability of GATK v. 2.7–4 UnifiedGenotyper to identify variants, we realigned reads from the reference isolate R265 back to the R265 genome after introducing 60 000 SNPs (corresponding to within VGII variation) and calculated the FDR [44]. Our alignment and SNP-calling approach were optimized for maximum specificity, which was necessary for characterizing microevolutionary differences. Specifically, we identified 59 578 (99.30%) true-positive SNPs, but only found 77 (0.13%) false-positive SNPs. For gene presence/absence polymorphisms, we counted all genes that had less than three times depth of coverage.

For our phylogenetic analysis, we extracted all positions that were called single base homozygous (reference or SNP) and polymorphic in one or more isolate in the 66 isolates (figure 1) encompassing 1 192 514 nuclear sites and 767 mitochondrial sites. We inferred the phylogeny of the isolates using RAxML v. 7.7.8 with the GTRCAT model and 1000 bootstrap replicates. For the PAML [37] selection analysis, we used the same tree building parameters on a subset of variants that were fixed in each of the isolates in one of six subclades, encompassing 647 792 sites.

Genes that localized to the mitochondria were identified using TargetP [56]. For our selection analysis, we calculated *d*_N_/*d*_S_ with yn00 of PAML [37] implementing the Yang and Nielsen method [57] on every gene in each of the six subclades (2a, 2b, 2c, 2x, 3a, 3b) using only fixed differences. For VGIIa, we used Codeml of PAML [37], implementing the BSM A (model  =  2, NSsites  =  2, fix_omega  =  0) compared with the null model (model  =  2, NSsites  =  3, fix_omega  =  1, omega  =  1) on every gene. Next, we calculated a Chi-squared test with 2 degrees of freedom for 2 × the log likelihood difference between the two compared models (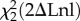) with BH multiple correction, and significance set at *q* < 0.01. Seven hundred and fourteen genes had values ranging from 1 to 2.25^−37^, whereas the remaining genes did not have values (e.g. owing to insufficient genetic distance).

## Supplementary Material

Summary of variant calling

## Supplementary Material

Summary of phenotypic analysis of Cryptococcus gattii strains.

## Supplementary Material

Genetic differences

## Supplementary Material

Presence/absence polymorphisms

## Data accessibility

The genome sequence and feature files for *C. gattii* R265 are available from GenBank (project accession number AAFP01000000).

All sequencing data are available from the SRA project accession SRP017762, and along with the phenotypic data, described in previous studies [16,24,52].
